# Co-morbidity of cervical incompetence with polycystic ovarian syndrome (PCOS) negatively impacts prognosis: A retrospective analysis of 178 patients

**DOI:** 10.1186/s12884-016-1094-6

**Published:** 2016-10-12

**Authors:** Yongqing Wang, Xunke Gu, Liyuan Tao, Yangyu Zhao

**Affiliations:** 1 Obstetrics & Gynecology Department, Peking University Third Hospital, Beijing, 100191 China; 2 Research Center of Clinical Epidemiology, Peking University Third Hospital, Beijing, 100191 China

**Keywords:** Cervical incompetence, Polycystic ovary syndrome, Preterm birth, Insulin resistance, Hyperandrogenism

## Abstract

**Background:**

Cervical incompetence is an important cause of miscarriage and premature birth and polycystic ovary syndrome is a heterogeneous endocrine disorder that is the most common cause of anovulatory infertility and eugonadotrophic hypogonadism. By now, it is still debated whether women with PCOS have an increased risk of miscarriage and there have been no studies about the pregnancy outcomes of cervical incompetence patients with PCOS.

**Methods:**

The following clinical data of cervical incompetence patients with/without PCOS who were treated between September 2006 and September 2013 were retrospectively analysed: onset gestational age, termination gestational age, pregnancy outcome, co-morbid insulin resistance (IR) in PCOS patients, the influence of IR, co-morbid hyperandrogenism (HA) in PCOS patients, and the influence of HA. The independent samples t-test and chi-square trend test were used to analyse the data.

**Results:**

A total of 178 singleton pregnancy cases with cervical incompetence were identified. The average onset gestational age was 23.9 ± 4.3 weeks, and the average termination gestational age was 32.5 ± 5.5 weeks. Of these 178 singleton pregnancy cases, 40 (22.5 %) ended in miscarriage, 82 (46.1 %) ended in preterm birth, and 56 (31.5 %) ended in term birth. Eighty cases (44.9 %) exhibited PCOS co-morbidity, and those cases had an average onset gestational age of 22.3 ± 3.8 weeks and an average termination gestational age of 31.2 ± 5.7 weeks, which were both significantly different from those of the non-PCOS group (both *P* < 0.001). Compared with the non-PCOS group (15.3 % miscarriage, 48.0 % preterm birth, and 36.7 % term birth), the PCOS group exhibited worse pregnancy outcomes (31.3 % miscarriage, 43.8 % preterm birth, and 25 % term birth) (*P* = 0.01). Among the 80 PCOS patients, 45 (56.3 %) exhibited co-morbid IR, and the IR group exhibited significantly worse pregnancy outcomes than the non-IR group (*P* = 0.03). Among the 80 PCOS patients, 54 cases (67.5 %) exhibited co-morbid HA, and there was no statistical difference on the pregnancy outcomes between the two groups. The multivariate logistic regression model revealed that PCOS was significantly correlated with miscarriage (OR: 3.72, 95 % CI: 1.37–10.13).

**Conclusions:**

The cervical incompetence patients with co-morbid PCOS exhibited earlier onset gestational ages, earlier termination gestational ages and worse pregnancy outcomes. For patients with co-morbid insulin resistance, the pregnancy outcomes were worse than expected.

## Background

Cervical incompetence is an important cause of miscarriage and premature birth and its definition is imprecise. It is sometimes defined as a biochemical and biomechanical problem (structural “weakness” due to acquired or inherent cervical tissue defects), sometimes as a clinical problem (the event/outcome of asymptomatic mid-trimester pregnancy loss), but usually the phrase is meant to encompass both [1]. The aetiology and pathogenesis of cervical incompetence remain unclear [2].

Cervical competence is now believed to change serially during the pregnancy process. Congenital dysplasia of the cervix, a lack of elastic fibres in the cervix, and a reduced collagen/smooth muscle ratio can lead to cervical incompetence. The causes of acquired cervical incompetence include mechanical injury, trauma, and biochemical factors. The length of the remaining cervix after conization is related to the incidence of cervical incompetence [3].

Although some women diagnosed with cervical incompetence actually have poor cervical integrity, the cervical anatomy of most such women is normal [4]. The generally accepted cause of cervical incompetence is premature cervical ripening, which leads to miscarriage/preterm birth due to subclinical infection, local inflammation, hormonal effects, and genetic factors [4].

Patients with cervical incompetence often do not experience contractions or obvious clinical symptoms before miscarriage or premature birth, which decreases the chance of providing treatment. Emergency cervical cerclage is sometimes performed in patients with dilation of the cervix, but infections and postoperative contractions with subsequent miscarriage often occur with this procedure [5].

Polycystic ovary syndrome (PCOS) is a heterogeneous endocrine disorder that is the most common cause of anovulatory infertility and eugonadotrophic hypogonadism [6–8]. The typical symptoms of PCOS include hyperandrogenism(HA), ovulation dysfunction, and polycystic ovaries. Approximately 50–70 % of PCOS cases are complicated by insulin resistance (IR). IR is a critical characteristic of metabolic syndrome [9], which is defined as insulin hyporeactivity and reduced absorption and utilization of glucose. Thus, in metabolic syndrome patients, insulin secretion increases, causing hyperinsulinemia and the exhaustion of the β cells in pancreatic islets [10]. Currently, IR is considered a basic pathological feature of PCOS, and higher concentrations of insulin can increase androgen levels and affect fertilization and implantation in PCOS patients [11].

Previous studies found that women with PCOS exhibited a clinically significant increased risk of pregnancy compared with controls [12]. Data which were not adjusted for BMI demonstrated that women with PCOS presented a 3–4-fold increased risk of developing pre-eclampsia, a 3-fold increased risk of gestational diabetes and 2-fold higher chance of premature delivery. But it is still debated whether women with PCOS have an increased risk of miscarriage compared with women without PCOS [12]. And by now, there have been no studies about the pregnancy outcomes of cervical incompetence patients with PCOS.

Previous studies found that androgens were crucial for cervical remodelling and myometrial ripening, but they failed to show a consistent relationship between either androgen excess or androgen deficiency and pregnancy outcomes. And the underlying mechanism by which androgens impact pregnancy outcomes has not been fully understood [13].

In this study, we retrospectively analysed the clinical data of cervical incompetence patients treated at the Department of Obstetrics and Gynecology, Peking University Third Hospital during the last 7 years to explore the clinical characteristics and pregnancy outcomes of cervical incompetence patients, particularly cervical incompetence patients with PCOS, IR and HA.

## Methods

### Data sources

The clinical data of patients who received routine prenatal care at our hospital and were diagnosed with cervical incompetence were collected between September 2006 and September 2013. This study was approved by the Ethics Committee of Peking University Third Hospital (No. 2014204). Written informed consent was not obtained from the participants because this was a retrospective study. All clinical records/information were anonymized and de-identified prior to analysis. The inclusion criterion was singleton pregnancy with cervical incompetence. The exclusion criteria were the following: i) dysplasia or malformation of the reproductive system (such as a unicornuate uterus, septate uterus, or double uterus), ii) infection, iii) multiple pregnancy, iv) a history of cervical conization, and v) a history of cervical incompetence. The enrolled patients were treated with bed rest, progesterone, or cerclage according to their gestational week, cervical length, and internal os width. We analysed the onset gestational ages (defined here as the gestational age at the onset of cervical incompetence), termination gestational ages and pregnancy outcomes of these patients. We divided the patients into PCOS and non-PCOS groups and compared their clinical characteristics. Then, we divided the patients with PCOS into IR and non-IR groups and compared their clinical characteristics. Similarly, we divided the patients with PCOS into HA and non-HA groups and compared their clinical characteristics.

The diagnosis of cervical incompetence depends on clinical symptoms and ultrasonic results [14, 15]. The diagnosis of PCOS was based on the Rotterdam 2003 criteria [16], which requires the presence of at least two of the following three features: i) oligo- and/or anovulation, ii) clinical and/or biochemical signs of hyperandrogenism, and iii) polycystic ovaries; the Rotterdam 2003 criteria also require the exclusion of other identifiable endocrine disorders, such as late-onset congenital adrenal hyperplasia, hyperprolactinemia, thyroid dysfunction, and neoplastic androgen secretion. IR level was evaluated using the Homeostatic Model Assessment (HOMA-IR) index calculated before pregnancy [17],HOMA-IR = fasting insulin(lU/ml) × fasting glucose(mmol/L)/22.5,and if HOMA-IR > 3.6, IR was diagnosed. HA before pregnancy is defined as excessive levels of androgens and their associated effects, except adrenal hyperplasia. Miscarriage was defined as foetal delivery at <28 weeks of gestational age in this study, and preterm delivery was defined as foetal delivery at ≥28 weeks but <37 weeks of gestational age.

### Data analysis

The data were captured from the Hospital Information System and were managed by Microsoft Excel 2013. All analyses were performed using SPSS version 18.0 (SPSS Inc., Chicago, IL, USA). The quantitative parameters that were normally distributed were expressed as χ^2^ values and were tested by the independent samples t test. Proportions were used to describe the characteristics and pregnancy outcomes of the patients. The Pearson’s Chi-square test and Chi-square trend test were applied to examine proportions between groups. The factors that may have affected pregnancy outcomes were analysed by multivariate logistic regression. A 2-sided *p* value of less than 0.05 was considered significant.

## Results

A total of 31,466 patients delivered at Peking University Third Hospital during the 7-year study period. Among these patients, 178 were diagnosed with cervical incompetence (0.6 %); the age range of those patients was 21 to 40 years, with an average age of 31.0 ± 3.9 years. Of the 158 patients (88.8 %) that were primiparous, 45 (25.3 %) had undergone assisted reproductive technology (ART) treatment and 30 (16.9 %) had co-morbid diabetes mellitus (DM)/gestational diabetes mellitus (GDM). We divided the patients into PCOS (80/178) and non-PCOS (98/178) groups. Age, parity and the frequencies of ART treatment and DM/GDM comorbidity were not significantly different between the two groups (Table 1).

**Table 1:** The basic characteristics of the 178 included patients

	Total (*n* = 178)	CI		
		PCOS(*n* = 80)	Non-PCOS(*n* = 98)	*p*
Age $\left(\overline{x}\pm s\right)$	31.0 ± 3.9	31.5 ± 4.1	30.6 ± 3.8	0.11^a^
Parity (n/%)				
Primiparity	158(88.8)	72(90.0)	86(87.8)	0.64^b^
Multiparity	20(11.2)	8(10.0)	12(12.2)	
Received ART (n/%)				
Yes	45(25.3)	24(30.0)	21(21.4)	0.19^b^
No	133(74.7)	56(70.0)	77(78.6)	
Co-morbid DM/GDM (n/%)				
Yes	30(16.9)	17(21.3)	13(13.3)	0.16^b^
No	148(83.1)	63(78.7)	85(86.7)	
BMI	24.2 ± 3.4	26.8 ± 3.0	22.1 ± 2.0	<0.01

^a^Independent samples t test

^b^ Chi-square test

For the 178 included patients, the average onset gestational age was 23.9 ± 4.3 weeks, and the average termination gestational age was 32.5 ± 5.5 weeks. Forty pregnancies (22.5 %) ended in miscarriage, 82 (46.1 %) ended in preterm birth and 56 (31.5 %) ended in term birth. In the PCOS group, the average onset gestational age and termination gestational age were significantly lower (*P* < 0.01), and the pregnancy outcome was significantly worse (*P* < 0.05) than in the non-PCOS group (Table 2). The rate of cervical cerclage in the PCOS group and non-PCOS group was 56.3 % and 44.9 % respectively, and there was no statistical difference (*P* > 0.05). (Table 2)

**Table 2:** The pregnancy outcomes of the 178 included patients

	Total (*n* = 178)	CI		
		PCOS(*n* = 80)	Non-PCOS(*n* = 98)	*p*
Onset gestational age $\left(\overline{x}\pm s\right)$	23.9 ± 4.3	22.3 ± 3.8	25.1 ± 4.1	<0.01^a^
Termination gestational age $\left(\overline{x}\pm s\right)$	32.5 ± 5.5	31.2 ± 5.7	33.6 ± 5.1	<0.01^a^
Pregnancy outcomes (n/%)				
Miscarriage	40(22.5)	25(31.3)	15(15.3)	0.03^b^
Preterm birth	82(46.0)	35(43.2)	47(48.0)	
Term birth	56(31.5)	20(25.0)	36(36.7)	
Pregnancy outcomes ^2^ (n/%)				
Miscarriage (less than 24 weeks)	16(22.5)	12(15.0)	4(4.1)	0.02^b^
Preterm birth	106(46.1)	48(60.0)	58(59.2)	
Term birth	56(31.5)	20(25.0)	36(36.7)	
Cervical cerclage	89(50.0)	45(56.3)	44(44.9)	>0.05

^a^Independent samples t test

^b^ Chi-square test

^1^Miscarriage was defined as foetal delivery at <28 weeks of gestational age

^2^ Miscarriage was defined as foetal delivery at <24 weeks of gestational age

Women in PCOS group were further divided into co-morbid IR and non-IR groups for comparison. Among the 80 PCOS patients, 45 cases (56.3 %) had the co-morbidity of IR. For the IR group, the onset gestational age and termination gestational age were significantly lower (*P* = 0.03, 0.01), and the pregnancy outcome was significantly worse compared with the non-IR group (*P* = 0.03) (Table 3). similarly, women in PCOS group were further divided into co-morbid HA and non-HA groups for comparison. Among the 80 PCOS patients, 54 cases (67.5 %) exhibited co-morbid HA, and there was no statistical difference on the pregnancy outcomes between the two groups (*P* = 0.22) (Table 4).

**Table 3:** Pregnancy outcomes of the PCOS group

	PCOS + IR (*n* = 45)	PCOS + non-IR(*n* = 35)	*p*
Onset gestational age $\left(\overline{x}\pm s\right)$	21.4 ± 3.6	23.3 ± 3.9	0.03^a^
Termination gestational age $\left(\overline{x}\pm s\right)$	29.8 ± 5.9	33.0 ± 5.0	0.01^a^
Pregnancy outcomes (n/%)			
Miscarriage	19(42.2 %)	6(17.1 %)	0.03^b^
Preterm birth	17(37.8 %)	18(51.4 %)	
Term birth	9(20.0 %)	11(31.4 %)	

^a^Independent samples t test

^b^ Chi-square trend test

**Table 4:** Pregnancy outcomes of the PCOS group

	PCOS + HA (*n* = 54)	PCOS + non-HA (*n* = 26)	*p*
Onset gestational age $\left(\overline{x}\pm s\right)$	22.2 ± 3.4	22.4 ± 4.6	0.80
Termination gestational age $\left(\overline{x}\pm s\right)$	31.0 ± 6.0	31.8 ± 5.3	0.56
Pregnancy outcomes (n/%)			
Miscarriage (less than 28)	19(35.2 %)	6(23.1 %)	0.22
Preterm birth	20(37.0 %)	15(57.7 %)	
Term birth	15(27.8 %)	5(19.2 %)	

^a^Independent samples t test

^b^ Chi-square trend test

To detect whether there were potential confounding factors in our study, a multivariate logistic regression analysis was conducted with miscarriage as the dependent variable and PCOS, DM/GDM, ART, age, BMI and onset gestational age as the independent variables. As shown in Table 5, only PCOS had a significant effect on bad pregnancy outcomes (miscarriage) (OR = 3.02, 95 % CI:1.07–8.48).

**Table 5:** Results of different multivariate logistic regression models performed to determine the relationship between PCOS and miscarriage

	Crude OR		Adjusted OR^1^		Adjusted OR^2^	
	OR	95%CI	OR	95%CI	OR	95%CI
PCOS	2.52^*^	1.22–5.20	3.72^*^	1.37–10.13	3.02^*^	1.07–8.48
DM/GDM	1.06	0.15–2.69	0.94	0.36–2.51	0.87	0.33–2.30
ART	1.36	0.62–2.98	1.23	0.54–2.80	1.49	0.65–3.45
Age		-	0.98	0.89–1.07	0.95	0.86–1.04
BMI		-	0.92	0.80–1.06	0.95	0.82–1.09
Onset gestational Age		-	-	-	0.90^*^	0.81–0.99

Model 1 adjusted for age and BMI by multivariate logistic regression, model 2 adjusted for age, BMI and onset gestational age by multivariate logistic regression

* indicated *p* < 0.05

## Discussion

The present retrospective study revealed that patients with cervical incompetence co-morbid with PCOS exhibited earlier onset and termination gestational age as well as worse pregnancy outcomes than non-PCOS patients. PCOS patients with an IR co-morbidity exhibited earlier onset and termination gestational age. In addition, PCOS was significantly correlated with miscarriage, whereas DM/GDM, ART, age and BMI were not.

At present, there is no standard for the diagnosis and treatment of cervical incompetence. The diagnosis of cervical insufficiency is challenging due to the lack of objective findings and clear diagnostic criteria for this disease. The diagnosis is based on a history of painless cervical dilation after the first trimester with subsequent expulsion of the pregnancy in the second trimester, typically before 24 weeks of gestation, and a lack of contractions, labour or any other clear pathology (e.g., bleeding, infection, etc.). In our study, all patients were hospitalized because of painless cervical shortening or dilation. Although the onset gestational age of some patients was greater than 24 weeks, all patients presented with painless cervical dilation without contractions, bleeding or any symptoms of infection and did not have threatened miscarriage or threatened premature birth at admission. According to the literature, the morbidity of cervical incompetence in pregnant women is 0.05 % ~ 1 % [18]. Our study, which excluded patients with multiple pregnancies and reproductive system malformation, revealed an incidence of cervical incompetence of 0.56 %.

Previous studies have reported that pregnant women with PCOS are susceptible to perinatal complications regardless of the definition of perinatal complications used, including elevated risk of gestational diabetes, pregnancy-induced hypertension, pre-eclampsia, preterm delivery and Caesarean section [19, 20]. Consistent with the findings of those studies, our results revealed that the co-morbidity of PCOS in cervical incompetence patients had exacerbating effects with respect to the onset and termination gestational ages and pregnancy outcomes. Furthermore, our logistic regression analysis confirmed the correlation of PCOS with miscarriage. Our results were also consistent with those of Farinelli et al. [21], who found no correlation between BMI and cervical incompetence. For patients with PCOS, cervical incompetence presents in an earlier gestational week, suggesting that early detection of cervical length may help in the early diagnosis and treatment of cervical incompetence. But unfortunately, in this study, some data of the cervical length was missing, and this is a limitation.

Previous studies have investigated the high incidence of miscarriage in women with PCOS and identified various aspects of PCOS, including hyperinsulinemia and other endocrine disorders, that are involved in the correlation of PCOS with miscarriage. In our study, PCOS patients with an IR co-morbidity exhibited earlier onset gestational age and earlier termination gestational age, indicating that these women warrant more aggressive treatment to prevent cervical incompetence, such as beginning monitoring at an earlier gestational age and increasing the monitoring frequency. We speculate that the high incidence of miscarriage in PCOS patients with the co-morbidity of IR maybe due to the following factors: IR may aggravate PCOS conditions, and IR may induce a glucose metabolism disorder that directly affects cervical incompetence, resulting in an adverse outcome. More research is needed to clarify the mechanisms through which IR increases the incidence of miscarriage.

In this study, we found no statistical difference on the pregnancy outcomes between the HA and non-HA groups. But the limitation is that we failed to get the data of androgen concentrations throughout gestation. Previous studies have found that androgens were crucial for cervical remodelling and myometrial ripening which may lead to preterm birth, but they failed to show a consistent relationship between either androgen excess or androgen deficiency and pregnancy outcomes. Further investigation of the underlying mechanisms of androgen action on cervical remodelling and myometrial contractility is needed.

Our study included 178 patients with cervical incompetence who delivered at our hospital during the past 7 years. Due to the long study duration and large numbers of cases, our findings are representative of the Chinese population. Our study has the following limitations: first, clinical data showed that all the 80 PCOS patients had examinations before pregnancy and they were diagnosed as PCOS according to the Rotterdam 2003 criteria, and the remaining 98 patients were diagnosed as non-PCOS with regular menstrual cycles and no clinical symptoms of androgen excess. There may be a little bias. Second, our hospital does not routinely screen for IR in women without PCOS; therefore, we did not have data regarding IR in non-PCOS patients. Thus, we cannot conclude whether the effect of IR on pregnancy outcomes is dependent on PCOS. Third, according to our medical treatment conditions, miscarriage is defined as foetal delivery at <28 weeks of gestational age in China, which is different from that in the developed countries such as America (<24 weeks) and England (<20 weeks). Compared with the non-PCOS group, the miscarriage rate (<28 weeks and <24 weeks) in the PCOS group was significantly higher. Due to few samples, the data of miscarriage (<20 weeks) rate was not calculated. Fourth, the estimated odds ratios (ORs) in the multistate analysis maybe have bias due to relatively small sample number.

In summary, it is very important for pregnant women with PCOS to undergo regular ultrasound examinations. In recent years, patients at our hospital with histories of cervical incompetence, recurrent miscarriages at middle gestational ages and cervical cone biopsy surgery have received two types of treatment. One is prophylactic cervical cerclage at the beginning of the middle gestational weeks, and the other is repetitive ultrasound examinations for cervical length every 2–4 weeks from the beginning of the third gestational month. For patients lacking a history of any of those disorders, we typically monitor cervical length via routine ultrasound examinations performed at 11–14 weeks, 21–24 weeks and 28–32 weeks of pregnancy. Based on the findings of our study, we suggest regularly monitoring cervical length during pregnancy for PCOS patients to achieve an early diagnosis and early treatment of cervical incompetence. Obviously this monitoring strategy will be more expensive and will require more medical resources, so cost-benefit and cost effectiveness studies are necessary.

## Conclusion

We found that patients with cervical incompetence and PCOS exhibited a poor prognosis and that the prognosis was even worse for patients with the additional co-morbidity of IR. Thus, in the clinic, early monitoring and early therapy will help to improve the prognosis of patients with both PCOS and IR. Furthermore, treatment of cervical incompetence requires both obstetric and endocrine input. We hope that our research will attract the attention of brilliant scientists in other subject areas and will help direct future relevant studies.
